# The association between thyroid function biomarkers and attention deficit hyperactivity disorder

**DOI:** 10.1038/s41598-020-75228-w

**Published:** 2020-10-26

**Authors:** Diana Albrecht, Till Ittermann, Michael Thamm, Hans-Jörgen Grabe, Martin Bahls, Henry Völzke

**Affiliations:** 1 Institute for Community Medicine, University Medicine Greifswald, 17475 Greifswald, Germany; 2 Leibniz Institute for Plasma Science and Technology (INP), 17489 Greifswald, Germany; 3 German Centre for Cardiovascular Research (DZHK), Partner-Site Greifswald, 17475 Greifswald, Germany; 4 Department of Epidemiology and Health Monitoring, Robert Koch Institute, 13353 Berlin, Germany; 5 Department of Psychiatry and Psychotherapy, University Medicine Greifswald, 17475 Greifswald, Germany; 6 German Centre for Neurodegenerative Disease (DZNE), Partner-Site Greifswald, 17475 Greifswald, Germany; 7 Department of Internal Medicine B, University Medicine Greifswald, 17475 Greifswald, Germany

**Keywords:** Thyroid diseases, ADHD, Paediatric research

## Abstract

The relation between thyroid function biomarkers and attention deficit hyperactivity disorder (ADHD) in children and adolescents is currently unclear. Cross-sectional data from the German Health Interview and Examination Survey for Children and Adolescents (KiGGS Baseline) was analyzed to assess the association between thyroid function biomarkers and ADHD in a population-based, nationally representative sample. The study cohort included 11,588 children and adolescents with 572 and 559 having an ADHD diagnosis or symptoms, respectively. ADHD symptoms were assessed through the *Inattention/Hyperactivity* subscale of the Strength and Difficulties Questionnaire. ADHD diagnosis was determined by a physician or psychologist. Serum thyroid stimulating hormone (TSH), free triiodothyronine (fT3), and free thyroxine (fT4) concentrations were determined enzymatically. Adjusted regression models were used to relate serum TSH, fT3, and fT4 with risk for ADHD diagnosis or symptoms. In children, a 1 mIU/l higher TSH was related to a 10% lower risk (odds ratio [OR] 0.90; 95% confidence interval [CI] 0.81–1.00) of ADHD diagnosis. We found a significant positive association between fT3 and continuously assessed ADHD symptoms in children (β 0.08; 95% CI 0.03–0.14). Our results suggest that physical maturity may influence the association between thyroid function biomarkers and risk for ADHD.

## Introduction

Attention deficit hyperactivity disorder (ADHD) is one of the most common neurodevelopmental disorders in children and is showing an increasing prevalence^1^. Symptoms are characterized by difficulties with concentration and attention, with controlling behavior, and hyperactivity^2^. ADHD affects approximately 7.1% of children and adolescents worldwide^3^ exceeding previous estimates of 5.3%^4^, and persists into adulthood in 65% of cases^5^.

ADHD shows considerable clinical heterogeneity in terms of symptom severity, admixture of symptoms, developmental course, and comorbidities. These parameters are influenced by inherited and non-inherited factors. Genetic variants (i.e. single nucleotide polymorphisms) are likely to be involved in the pathophysiology of ADHD^6^. However, their effect sizes are rather small. Interestingly, twin and adoption studies^7–9^ found a high mean heritability of 0.77. Endocrine disorders also influence ADHD susceptibility. Specifically, environmental toxins in-utero or early childhood such as exposure to lead, organophosphate pesticides, and polychlorinated biphenyls are risk factors for ADHD^10^. ADHD susceptibility is not influenced by nutritional deficiencies, nutritional surpluses (e.g. sugar and artificial food colorings), and low or high Immunoglobulin G (IgG) foods^11^. Further evidence suggests that there is no causal association between maternal smoking during pregnancy and offspring ADHD^12^. While a recent meta-analysis provided evidence for an association between maternal smoking and offspring ADHD ^13^, the authors also reported significant heterogeneity between studies (I^2^ = 79.2%, *p* < 0.01).

Maternal thyroid dysfunction may adversely affect fetal brain development. Maternal (overt) hyperthyroidism^14,15^, high thyroid stimulating hormone (TSH) concentration during pregnancy^16^, and maternal autoimmune thyroiditis^17^ in early pregnancy increase the offspring’s risk for ADHD. Though there is an association between maternal thyroid dysfunction and offspring ADHD^18^, the knowledge on the association between iodine intake in pregnancy and offspring’s risk of diagnosed ADHD is sparse. A large Norwegian cohort study did not find a significant association with maternal iodine intake and child ADHD diagnosis but with symptoms^19^. This is supported by a large Danish cohort study. Inadequate maternal iodine intake and increased risk of ADHD symptoms in children is suggested by previous research^14^.

As synthesis and secretion of thyroid hormones are regulated by a negative feedback system that involves the hypothalamus, pituitary and thyroid gland (the HPT axis)^20^, thyroid function becomes important for the cognitive development in children and adolescents^21^. As individuals with hyperthyroidism may have ADHD-like symptoms (e.g. anxiety, nervousness, irritability, and physical hyperactivity)^22^, several studies investigated the association between thyroid dysfunction and ADHD in children. Zader et al. concluded that children with hyperthyroidism have an ADHD prevalence ratio of 1.7 compared to children without hyperthyroidism. Even more notably, in 40% of cases, the mental health diagnosis antedated the hyperthyroidism by 90 days, with ADHD being diagnosed before hyperthyroidism in 68.3% of cases^23^. Even within the normal range, TSH concentrations in the upper quartile were positively associated with ADHD symptoms in healthy children^24^. In adolescents, on the other hand, thyroid disorders were not related with ADHD^25^. Early studies with clinically referred ADHD children and adolescents were inconclusive with regards to thyroid dysfunction. Some supported a link between thyroid function abnormalities and the cognitive-behavioral manifestations of ADHD^26^, while others did not^27^. Hence, previous investigations showed large heterogeneity, which may be partly due to the relatively small sample sizes between 150^25,27^ and 350^24,26^ evaluated ADHD cases.

We associated TSH and circulating thyroid hormones with the risk of ADHD using cross-sectional data from the large-scale population-based German Health Interview and Examination Survey for Children and Adolescents (KiGGS Baseline). Specifically, we investigated first how serum TSH, free triiodothyronine (fT3), and free thyroxine (fT4) were related to ADHD symptoms as well as ADHD diagnosis. We adjusted our regression models with several covariates previously identified in the literature to be associated with thyroid function and ADHD. We included sex since male children are three times more likely to have ADHD compared to female children. In adulthood men and women are equally likely to have ADHD^28^. In line with this finding, recent research suggested that changes in the symptom profile of ADHD, with hyperactivity emerging in childhood for males but having later onset at the time of adolescence for females, may contribute to this pattern^29^. We also included body mass index (BMI) because previous meta-analyses found a positive association between ADHD and overweight or, in extreme cases, obesity^30,31^. ADHD has also been found to be associated significantly with low birth weight and prematurity in several cohort studies and meta-analyses^32,33^. Since maternal smoking is a risk factor for lower birth weight and preterm birth^34,35^, this covariate was also used along with weight at birth in our regression analysis. In summary, for our regression analysis we included offspring’s sex and birth weight, age, BMI and maternal smoking as covariates.

## Results

Tables 1 and 2 show the characteristics of the study population stratified by ADHD diagnosis and ADHD symptoms. In our population a total of 420 children and 152 adolescents were diagnosed with ADHD while 486 children and 73 adolescents showed ADHD symptoms.

**Table 1:** Characteristics of the study population by ADHD diagnosis cases.

	Children (tanner stage I–III)				Adolescents (tanner stage IV–V)			
	ADHD diagnosis	Control	Total	*p*	ADHD diagnosis	Control	Total	*p*
n	420	8265	8685		152	2751	2903	
TSH, mIU/L	2.1 (1.6; 3.0)	2.3 (1.7; 3.0)	2.3 (1.7; 3.0)	0.007	1.9 (1.3; 2.7)	1.8 (1.3; 2.5)	1.8 (1.3; 2.5)	0.54
fT3, pmol/L	6.1 (5.7; 6.7)	6.2 (5.7; 6.7)	6.2 (5.7; 6.7)	0.31	5.9 (5.4; 6.7)	5.7 (5.1; 6.3)	5.7 (5.1; 6.3)	0.001
fT4, pmol/L	18.1 (16.4; 19.6)	18.2 (16.8; 19.8)	18.2 (16.7; 19.8)	0.02	17.7 (16.2; 19.4)	18.1 (16.5; 19.9)	18.1 (16.5; 19.9)	0.18
Low TSH^a^, n (%)	13 (3.10)	146 (1.77)	159 (1.83)		1 (0.66)	17 (0.62)	18 (0.62)	
High TSH^a^, n (%)	6 (1.43)	242 (2.93)	248 (2.86)		10 (6.58)	133 (4.83)	143 (4.93)	
Low fT3^ a^, n (%)	27 (6.43)	358 (4.33)	385 (4.43)		5 (3.29)	87 (3.16)	92 (3.17)	
High fT3^a^, n (%)	11 (2.62)	157 (1.90)	168 (1.93)		3 (1.97)	65 (2.36)	68 (2.34)	
Low fT4^a^, n (%)	0 (0)	61 (0.74)	61 (0.70)		1 (0.66)	8 (0.29)	9 (0.31)	
High fT4^a^, n (%)	26 (6.19)	878 (10.62)	904 (10.41)		7 (4.61)	221 (8.03)	228 (7.85)	
Males	336 (80.0)	4129 (49.96)	4465 (51.41)		120 (78.95)	1272 (46.24)	1392 (47.95)	
Age, years	10.6 (8.8; 12.5)	8.8 (6.1; 11.5)	9.0 (6.3; 11.6)	< 0.001	15.6 (14.4; 16.7)	15.6 (14.4; 16.7)	15.6 (14.4; 16.7)	0.69
BMI, kg/m^2^	17.3 (15.9; 19.6)	16.6 (15.3; 18.9)	16.7 (15.3; 19.0)	< 0.001	21.5 (19.3; 25.2)	21.0 (19.3; 23.4)	21.0 (19.3; 23.5)	0.13
Weight at birth, g	3380 (3000; 3700)	3410 (3080; 3750)	3410 (3080; 3740)	0.03	3370 (3040; 3700)	3380 (3070; 3700)	3380 (3070; 3700)	0.95
**Maternal characteristics**								
Gestational week at birth	39 (38; 40)	40 (39; 40)	40 (39; 40)	0.008	40 (38; 40)	40 (39; 40)	40 (39; 40)	0.08
Gestational smoking n (%)	95 (23.34)	1321 (16.18)	1416 (16.52)		32 (21.77)	441 (16.28)	473 (16.57)	
Socioeconomic status	10 (8; 13)	11 (9; 15)	11 (9; 15)	< 0.001	11 (8; 14)	11 (9; 15)	11 (8; 15 )	0.05

Continuous data expressed as median and 25th/75th percentiles; nominal data expressed as total numbers and percentages; significance levels for continuous data was determined with the test for difference in median.

*TSH* thyroid stimulating hormone, *fT3* free triiodothyronine, *fT4* free thyroxine, *BMI* body mass index.

^a^Defined according to Kratzsch et al^36^.

**Table 2:** Characteristics of the study population by ADHD symptoms cases.

	Children (tanner stage I–III)				Adolescents (tanner stage IV–V)			
	ADHD symptoms	Control	Total	*p*	ADHD symptoms	Control	Total	*p*
n	486	7754	8240		73	2667	2740	
TSH, mIU/L	2.4 (1.8; 3.1)	2.3 (1.7; 3.0)	2.3 (1.7; 3.0)	0.07	1.7 (1.2; 2.3)	1.8 (1.3; 2.5)	1.8 (1.3; 2.5)	0.40
fT3, pmol/L	6.2 (5.7; 6.8)	6.2 (5.7; 6.7)	6.2 (5.7; 6.7)	0.66	6.0 (5.3; 6.7)	5.7 (5.1; 6.3)	5.7 (5.1; 6.3)	0.006
fT4, pmol/L	18.1 (16.7; 19.8)	18.2 (16.8; 19.8)	18.2 (16.8; 19.8)	0.38	17.8 (16.4; 19.6)	18.1 (16.5; 19.9)	18.1 (16.5; 19.9)	0.37
Low TSH^a^, n (%)	7 (1.44)	139 (1.79)	146 (1.77)		0 (0)	17 (0.64)	17 (0.62)	
High TSH^a^, n (%)	16 (3.29)	226 (2.91)	242 (2.94)		5 (6.85)	127 (4.76)	132 (4.82)	
Low fT3^ a^, n (%)	16 (3.29)	341 (4.40)	357 (4.33)		1 (1.37)	86 (3.22)	87 (3.18)	
High fT3^a^, n (%)	8 (1.65)	148 (1.91)	156 (1.89)		3 (4.11)	61 (2.29)	64 (2.34)	
Low fT4^a^, n (%)	3 (0.62)	58 (0.75)	61 (0.74)		0 (0%)	8 (0.30)	8 (0.29)	
High fT4^a^, n (%)	47 (9.67)	828 (10.68)	875 (10.62)		6 (8.22)	215 (8.06)	221 (8.07)	
Males, n (%)	296 (60.91)	3821 (49.28)	4117 (49.96)		44 (60.27)	1222 (45.82)	1266 (46.20)	
Age, years	8.2 (5.6; 10.6)	8.9 (6.2; 11.6)	8.8 (6.1; 11.5)	< 0.001	15.0 (14.3; 16.4)	15.6 (14.4; 16.8)	15.6 (14.4; 16.7)	0.04
BMI, kg/m^2^	16.4 (15.1; 18.2)	16.7 (15.3; 19.0)	16.6 (15.3; 19.0)	0.007	21.0 (19.2; 23.1)	21.0 (19.3; 23.4)	21.0 (19.3; 23.4)	0.80
Weight at birth, g	3350 (3000; 3650)	3420 (3090; 3750)	3410 (3080; 3750)	0.003	3370 (3020; 3800)	3380 (3070; 3700)	3380 (3070; 3700)	0.92
**Maternal characteristics**								
Gestational week at birth	40 (38; 40)	40 (39; 40)	40 (39; 40)	0.03	40 (38; 40)	40 (39; 40)	40 (39; 40)	0.36
Gestational smoking, n (%)	143 (29.9)	1175 (15.3)	1318 (16.19)		21 (30.43)	419 (15.94)	440 (16.31)	
Socioeconomic status	10 (7; 12)	11 (9; 15)	11 (9; 15)	< 0.001	11 (8; 13)	11 (9;15)	11 (9;15)	0.09

Continuous data expressed as median and 25th/75th percentiles; nominal data expressed as total numbers and percentages; significance levels for continuous data was determined with the test for difference in median.

*TSH* thyroid stimulating hormone, *fT3* free triiodothyronine, *fT4* free thyroxine, *BMI* body mass index.

^a^Defined according to Kratzsch et al^36^.

TSH and ft4 concentrations were significantly lower in children with compared to children without ADHD diagnosis (Table 1). Adolescents with either ADHD diagnosis or ADHD symptoms had higher fT3 levels compared to controls. Very few study participants had TSH (n = 58, 0.005%), fT3 (n = 74; 0.006%) and fT4 (n = 90; 0.008%) concentrations outside the reference range defined by Kratzsch et al^36^.

Children in both groups, ADHD diagnosis and ADHD symptoms, were born during an earlier gestational week, weighted less at birth and had a lower socioeconomic status compared to controls. Children with an ADHD diagnosis had a higher BMI while children with ADHD symptoms had a lower BMI compared to controls. In adolescents no differences for BMI, gestational week of birth, birth weight and socioeconomic status were found.

The association of serum TSH, fT3 and fT4 for both groups, ADHD diagnosis and ADHD symptoms as categorical variable, is presented in Table 3. In children, a higher TSH concentration was associated with a lower risk for ADHD diagnosis. TSH was not related to ADHD symptoms in children or adolescents. Further, fT3 or fT4 were not significantly associated with ADHD diagnosis and ADHD symptoms as a categorical variable for neither children nor adults. When looking at ADHD symptoms as a continuous variable, fT3 was positively associated with ADHD symptoms in children (Table 4). We found no statistically significant relationship between TSH or fT4 with ADHD symptoms as a continuous variable for children or adolescents.

**Table 3:** Results for the linear regression analysis between thyroid function biomarkers and ADHD diagnosis and ADHD symptoms.

	ADHD diagnosis		ADHD symptoms	
	Categorical		Categorical	
	Children (tanner I–III)	Adolescents (tanner IV–V)	Children (tanner I–III)	Adolescents (tanner IV–V)
	OR (CI)	OR (CI)	OR (CI)	OR (CI)
TSH, mIU/L	0.90 (0.81; 1.00)*	1.05 (0.90; 1.23)	1.00 (1.00; 1.01)	1.00 (0.78; 1.28)
fT3, pmol/L	1.00 (0.88; 1.12)	1.03 (0.81; 1.30)	1.02 (0.92; 1.14)	1.16 (0.89; 1.51)
fT4, pmol/L	0.96 (0.91; 1.01)	0.94 (0.86; 1.03)	0.97 (0.92; 1.02)	0.99 (0.90; 1.09)

Presented are adjusted odds ratios (OR) with 95% Confidence Interval (CI). Models have been adjusted for sex, age, and weight at birth, and the following covariates: mother’s smoking habit during gestation and the current BMI z-score of the child/adolescent.

*TSH* thyroid stimulating hormone, *fT3* free triiodothyronine, *fT4* free thyroxine.

*p ≤ 0.05.

**Table 4:** Results for the linear regression analysis between thyroid function biomarkers and ADHD symptoms continuously assessed by the *inattention/hyperactivity* subscale.

	ADHD symptoms	
	Continuous	
	Children (tanner I–III)	Adolescents (tanner IV–V)
	β (CI)	β (CI)
TSH, mIU/L	0.00 (− 0.03; 0.04)	0.03 (− 0.10; 0.04)
fT3, pmol/L	0.08 (0.03; 0.14)*	0.07 (− 0.02; 0.16)
fT4, pmol/L	− 0.02 (− 0.04; 0.00)	− 0.02 (− 0.06; 0.01)

Presented are beta-coefficients and 95% confidence intervals (CI) for a one unit change in thyroid function biomarker. Models have been adjusted for sex, age, and weight at birth, and the following covariates: mother’s smoking habit during gestation and the current BMI z-score of the child/adolescent.

*TSH* thyroid stimulating hormone, *fT3* free triiodothyronine, *fT4* free thyroxine.

*p ≤ 0.05.

For sensitivity analyses, we excluded all children and adolescents with an already diagnosed thyroid disorder as well as those with TSH, fT3 or fT4 values outside the reference range as defined by Kratzsch et al.^36^ In this subgroup, we observed no significant associations of TSH, fT3 or fT4 with ADHD diagnosis or symptoms. In another sensitivity analysis, we excluded all obese children and adolescents. Here we found a strong trend for the association between TSH with ADHD diagnosis in children (*p* = 0.064). However, the effect size was similar to the one of the main analyses. Likewise, when excluding children with a birthweight below the gestational week specific 10th percentile the effect size for TSH on ADHD diagnosis did not change compared to the main analysis, but the p-value was 0.055. When excluding children born preterm, the effect size for the association between TSH and ADHD cases was markedly lower than in the main analysis (OR 0.93; 95% CI 0.83–1.04; *p* = 0.204). We have also tested interactions of TSH, fT3 and fT4 with sex on ADHD diagnosis and symptoms; however, we did not find any significant interactions (e.g. interactions for TSH-sex on ADH diagnosis was 0.759 in children and 0.595 in adolescents).

## Discussion

The present study investigated whether thyroid function biomarkers of children and adolescents are associated with ADHD diagnosis or symptoms. We demonstrated that higher serum TSH levels were associated with a lower risk for ADHD diagnosis in children but not adolescents. When analyzing ADHD symptoms as a continuous variable, fT3 was positively associated with ADHD symptoms in children. No relationship was found for fT4 with an ADHD diagnosis or ADHD symptoms in children or adolescents. Overall, our results support the notion that lower serum TSH may be related to a higher risk for ADHD in children but not adolescents. These findings agree with recent work by Villanger et al. who reported that ADHD risk appeared to be elevated among newborns with low TSH levels after analyzing TSH concentrations below 10 mIU/L in 405 ADHD cases and 1092 controls^37^.

Thyroid function can be determined either directly, by measuring thyroid hormones or indirectly, by assessing TSH concentrations, which inversely reflects the thyroid hormone concentration sensed by the pituitary^24^. Therefore, serum TSH measurements might offer a better sensitivity for detecting thyroid dysfunction in comparison to fT3 and fT4 testing. This is especially important in light of our results regarding the positive association between TSH and ADHD diagnosis in children.

Solid evidence points to thyroid function being pivotal for brain development^38–41^. As this holds true for all stages of brain development, maternal thyroid function is important for early brain development while childhood thyroid function is important for subsequent stages of the maturing brain^42,43^. Our analysis showed that although the prevalence of ADHD diagnosis remains approximately the same between children and adolescents, thyroid hormones are different. In children with an ADHD diagnosis, serum TSH and fT4 were lower. In adolescents with either an ADHD diagnosis or ADHD symptoms, fT3 was higher. Hence, serum TSH and fT3 may influence the risk for ADHD.

In our cohort, a 1 mIU/L higher TSH was related to a 10% lower risk for ADHD in children previously diagnosed with ADHD. These data may support the importance of TSH during early brain development, since these associations disappeared in adolescents. In addition, since we found no significant relations between TSH and fT4 with ADHD symptoms, our results suggest that these parameters may not be suitable for ADHD diagnosis in adolescents. In addition, it should be noted that the positive association of fT3 with ADHD symptoms might simply be measures of behavior at the high-end normal variation for the *Inattention/Hyperactivity* scale.

While earlier investigations explored the association between severe thyroid dysfunction and ADHD in children and adolescents, these cases, 224 in total, were excluded from our analysis. Thyroid function and ADHD symptoms may have changed significantly since the medication was initiated; therefore, it is difficult to assess the influence of present thyroid function when participants are on thyroid medication.

This investigation has several limitations. First, ADHD diagnosis was parent-reported after a previous diagnosis by a physician or psychologist and the age of the children/adolescents at time of diagnosis is unknown. We do acknowledge that when assessing ADHD symptoms, multi-informant rating scales are highly recommended and widely used to assist in decision-making^2,44^. Unfortunately, collecting additional information from teachers and/or relatives was unfeasible in this cohort study with more than 10,000 participants. Second, the use of stimulant medication (e.g. methylphenidate) in participants with an ADHD diagnosis was not assessed in the questionnaire. However, methylphenidate treatment had no sustained effects on thyroid function in pre-pubertal children with ADHD^45^. Third, although both hypo- and hyperthyroidism in mothers during pregnancy have been associated with a greater risk of ADHD in children, the cohort did not include information on thyroid status of the mother during pregnancy. Fourth, our significant findings may be driven by our large sample size (more than 10,000 children and adolescents) influencing our p-values. Fifth, a meta-analysis of studies with longitudinal follow-up of children with ADHD suggests that 35% of children recover whereas 50% meet criteria for ADHD in partial remission and 15% continue to meet full diagnostic criteria for ADHD^5,46^. Lastly, our cross-sectional study design cannot provide causal relationships between thyroid function biomarkers and ADHD diagnosis, therefore a longitudinal analysis should assess when and how thyroid dysfunction may contribute to the pathophysiology of ADHD.

Nonetheless, this analysis also has several strengths. Our study population consisted of a very large cohort of children and adolescents (n > 10,000) representative of the German youth. Specifically, the information on numerous potential confounders was available. For the assessment of ADHD symptoms of participants aged 3 to 11 years, a behavioral assessment was conducted during medical examination in addition to the Strengths and Difficulties Questionnaire (SDQ) score of the parents’ questionnaire. Moreover, a central laboratory was used to measure age-specific TSH, fT3, fT4 thereby reducing variability.

The various short- and long-term medical, social and health economic consequences of ADHD illustrate the high level of public health relevance of this disorder. Yet, possibilities of primary prevention are limited due to the high proportion of genetic factors in the etiology of ADHD. The association of thyroid dysfunction with neurodevelopmental disorders has always been of special interest because the brain is a target organ for thyroid hormones, and even small differences in neurobehavioral outcomes can have major public health consequences^47^. The association between TSH and ADHD diagnosis in children within the KiGGS Baseline study further highlights the significance to promote investigations to explore this interaction. Also, the lack of an association between TSH and ADHD diagnosis in adolescents points to important timing considerations for treatment options.

## Methods

### Participants and study design

KiGGS Baseline is based on a cross-sectional, nationally representative sample of children and adolescents 0–17 years of age with their main residence in Germany^48^. The sampling procedure followed a two-stage protocol developed in cooperation with the Centre for Survey Research and Methodology (ZUMA), Mannheim, Germany. First, to consider proportionately the population size according to degree of urbanization and geographic distribution in Germany, 167 communities were selected as primary sample units (PSUs), with a disproportionate number of PSUs in Berlin and East as well as West Germany, to represent these regions separately. At the second stage, an equal number of addresses (n = 24) per birth cohort was randomly selected (simple random sample) from local population registries within selected PSUs, eight weeks before the start of examinations. A final simple random sample was drawn at the Robert Koch Institute (RKI, Berlin, Germany), including a total of eight, nine, or ten children and adolescents per birth cohort, depending on community size. The total KiGGS Baseline sample included 28,299 children and adolescents. The ethics committee of the Charité/University Medicine Berlin approved the study.

Parents of eligible children and adolescents were contacted by letter and invited to participate in the survey. Following a random route plan, 167 PSUs were covered by four study teams within three years (May 19, 2003 to May 6, 2006). Personal contact was sought to those who had not responded to the invitation letter. A total of 17,641 children and adolescents participated in the study (response 66.6%). The parents of all participants gave informed written consent.

For the purpose of our study of the 17,641 KiGGS Baseline participants, children younger than three years were excluded (n = 2805) because of inadequate blood sample volume to measure thyroid function biomarkers. Further, we excluded 224 participants taking thyroid medication and 2871 participants with missing data for serum TSH levels or other co-variables leaving 11,588 participants for the analysis of thyroid function biomarkers and ADHD diagnosis. For the second regression analysis, assessing the relation between thyroid function biomarkers and ADHD symptoms, missing data was present for 36 participants. Further, the 572 ADHD diagnosed individuals were excluded in this analysis resulting in a final sample size of 10,980 children and adolescents.

### Interview and physical examination

The examination and interview component, which was tailored to age range, was carried out in a Standard Operating Procedure (SOP)-compliant, standardized manner. Each team involved in the study, which consisted of a physician and two examiners, attended a comprehensive training program explaining the guidelines set out in the SOP manuals before starting fieldwork. Follow-up training was regularly carried out during the data collection phase as required. Children’s age, sex, birth weight, gestational week at birth as well as maternal smoking during gestation (regularly, occasionally, never) and family’s socioeconomic status (SES), which was defined as “low”, “medium”, or “high” based on an established and validated index by Winkler and Stolzenberg^49^, were determined using computer-assisted parental interviews conducted by a study physician. This multidimensional aggregated SES index comprises the dimensions of parents’ education (school education and professional qualifications), income (net household income of all household members), and occupational status. In addition, a broad range of health information was collected from a self-administered questionnaire. These questionnaires were child age group-specific and administered to parents of all participants aged 0–17 years and in addition directly to participants aged 11–17 years. The parental questionnaire included a question on whether their child had been diagnosed with ADHD according to the established symptom profile (10th edition; ICD-10^50^) through a multi-informant assessment^2^. From the already frequently tested and validated Strengths and Difficulties Questionnaire (SDQ)^51^ the subscale *Inattention /Hyperactivity* was included in the parental questionnaire. To determine ADHD symptoms the cutoff value of the English standard random sample was used^52^, which is identical with the cut-off value of the German standardization^53^. The internal consistency of Subscale *Inattention/Hyperactivity* is α = 0.59 and varies from 0.52 (lowest value, age group 3–6 years) to 0.63 (highest value, age group 11–13 years)^54^. Although a multi-informant assessment was not feasible to be ascertained in the KiGGS study setting, an additional behavioral assessment was carried out independently from the parental *Inattention/Hyperactivity* assessment by the study team during the physical examination (n = 7,919) for the age group of 3- to 11-year-olds. The criteria for the behavioral assessment conducted by the study team were oriented on the three main symptoms of ADHD: inattention, restlessness, impulsivity. Resulting from the parental questionnaires, participants were categorized as ADHD diagnosis cases, if a doctor or psychologists had diagnosed ADHD previously. ADHD symptoms were determined as followed: participants age 3- to 11-years-old, who scored a sum value of ≥ 7 in the *Inattention/Hyperactivity* scale of the SDQ and in addition received a positive behavioral assessment from the study team, yet had no previous ADHD diagnosis, were assigned in this group. For participants older than 11 years, the SDQ subscale result determined the ADHD symptoms. Participants were assigned to the control group for our first round of analyses, if no previous ADHD diagnosis was attested. In our second round of analyses, the control group consisted of participants with no previous ADHD diagnosis and whose behavioral assessment and the SDQ subscale result pointed towards no ADHD symptoms.

Participant BMI was calculated by dividing body weight in kg by height in cm to the square, which was measured during the physical examination. The Tanner Scale was observed by the study examiner and used to describe physical development^55^. The Tanner stages one through five were assigned based on the participants’ external primary and secondary sex characteristics, such as breast size and development of pubic hair. Afterwards all participants were classified into two groups according to their Tanner status: children (stages one through three) and adolescents (stages four and five). The differentiation by physical development was chosen as there are substantial changes in TSH and thyroid hormone levels during childhood, in particular for fT3, which appear to relate to pubertal readiness^56^.

### Thyroid function biomarkers

A non-fasting blood sample was drawn during the appointment for the health interview and examination and processed within 45 minutes^57^. TSH, fT3, and fT4 were determined using electrochemiluminescence (Elecsys 2010, Roche Diagnostics, Mannheim, Germany). The inter-serial coefficients of variation (CVs) were 3.9% for TSH, 5.9% for fT3, and 5.3% for fT4. The difference in the median serum TSH level was marginal regardless of the timing of the sample (morning, 2.11 mU/L; afternoon, 2.12 mU/L; evening, 2.31 mU/L) and unlikely to influence the results. As all measurements were conducted in the same laboratory, higher or lower thyroid hormone levels were defined using age- and sex-specific reference ranges^36^.

### Statistics

The total study population was stratified by Tanner status (children and adolescents) and the presence of an ADHD diagnosis or ADHD symptoms, which resulted in four different groups. Continuous data were expressed as median and 25th/75th percentiles (Q1 and Q3, respectively). Nominal data are provided as total numbers and percentages. Differences between groups were calculated using Kruskal–Wallis (continuous variables) and Χ^2^ test (nominal variables), respectively. Associations of TSH, fT3 and fT4 with ADHD diagnosis as well as categorical ADHD symptoms were calculated using logistic regression models adjusted for sex, age, weight at birth, mother’s smoking habit during pregnancy and current BMI z-score of the child/adolescent. Linear regression models adjusted for the same confounders were used to analyze the association between TSH, fT3 and fT4 with continuous ADHD symptoms. To account for potential non-linear relationships we modeled the exposure variable continuously and tested potential non-linear transformations of the exposure variable by sophisticated statistical methods such as fractional polynomials or restricted cubic splines to account for potential non-linear relationships of TSH/fT3/fT4 with ADHD diagnosis and symptoms. In our analyses, we found no evidence for non-linear associations. A *p* < 0.05 was considered statistically significant. All statistical analyses were performed in Stata 14 (StataCorp. 2015. College Station, TX, USA).

### Ethics approval and consent to participate

The parents/legal guardians of all participants gave informed written consent. The Ethics Committee of the Charité/University Medicine Berlin approved the study. Hence, all methods were carried out in accordance with relevant guidelines and regulations.

## Supplementary information

Supplementary Information.

## Data Availability

The data that support the findings of this study are available from the Robert Koch Institute, Germany but restrictions apply to the availability of these data, which were used under license for the current study, and so are not publicly available. Data are however available from the authors upon reasonable request and with permission of the Robert Koch Institute.
