# Supplementary material for: The association between thyroid function biomarkers and attention deficit hyperactivity disorder
Source: Sci Rep. 2020 Oct 26;10:18285. doi: 10.1038/s41598-020-75228-w (PMC7588469; doi:10.1038/s41598-020-75228-w)
Supplement: Supplementary file 1 — Supplementary Information. [file 41598_2020_75228_MOESM1_ESM.docx]

**The association between thyroid function biomarkers and attention deficit hyperactivity disorder**

Diana Albrecht^1,2^*, MPA, Till Ittermann^1, 3^, PhD, Michael Thamm ^4^,

Hans-Jörgen Grabe ^5, 6^, MD, PhD, Martin Bahls ^3, 7^, PhD, Henry Völzke^1, 3^, MD, PhD

**Affiliations**:

^1^ Institute for Community Medicine, University Medicine Greifswald, 17475 Greifswald, Germany

^2^ Leibniz Institute for Plasma Science and Technology (INP), 17489 Greifswald, Germany

^3^ German Centre for Cardiovascular Research (DZHK), partner-site Greifswald, 17475 Greifswald, Germany

^4^ Robert Koch Institute, Berlin, Germany

^5^ Department of Psychiatry and Psychotherapy, University Medicine Greifswald, 17475 Greifswald, Germany

^6^ German Centre for Neurodegenerative Disease (DZNE), Partner-site Greifswald, 17475 Greifswald, Germany

^7^ Department of Internal Medicine B, University Medicine Greifswald, 17475 Greifswald, Germany

[*diana.albrecht@uni-greifswald.de](mailto:*diana.albrecht@uni-greifswald.de)

In addition to our analyses in the main manuscript, we specifically investigated how serum TSH, free triiodothyronine (fT3), and fT4 outside the reference range were associated with ADHD diagnosis and ADHD symptoms in children and adolescents. We found that children with TSH levels above the reference range had 68 % lower risk for ADHD diagnosis (OR: 0.32; 95% CI: 0.10 to 0.98). Circulating thyroid hormone concentrations below or above the reference concentration were not associated with confirmed or suspected ADHD. However, the number of children and adolescents with thyroid function biomarkers outside the reference range was relatively small. Thus, this analysis may be limited by reduced statistical power to detect and validate any association between ADHD and thyroid function biomarkers concentration on either end of the spectrum.

|  | **ADHD diagnosis** | | | |  | **ADHD symptoms** | | | |
| --- | --- | --- | --- | --- | --- | --- | --- | --- | --- |
|  | Children (Tanner I-III) | | Adolescents (Tanner IV-V) | |  | Children (Tanner I-III) | | Adolescents (Tanner IV-V) | |
|  | N | OR (CI) | N | OR (CI) |  | N | OR (CI) | N | OR (CI) |
| **low TSH, mIU/L** | 13 | 1.04 (0.53; 2.02) | 1 | 2.21 (0.25; 19.68) |  | 7 | 0.45 (0.15; 1.35) | 0 | - |
| **high TSH, mIU/L** | 6 | 0.32 (0.10; 0.98)* | 10 | 1.27 (0.58; 2.78) |  | 16 | 1.07 (0.58; 1.98) | 5 | 1.12 (0.40; 3.15) |
| **low fT3, pmol/L** | 27 | 1.39 (0.87; 2.23) | 5 | 2.42 (0.88; 6.66) |  | 16 | 0.59 (0.34; 1.03) | 1 | 0.23 (0.03; 1.75) |
| **highfT3, pmol/L** | 11 | 0.55 (0.23; 1.34) | 3 | 0.52 (0.14; 1.88) |  | 8 | 0.76 (0.34; 1.69) | 3 | 1.77 (0.52; 6.08) |
| **low fT4, pmol/L** | 0 | - | 1 | - |  | 3 | 1.47 (0.36; 6.05) | 0 | - |
| **highfT4, pmol/L** | 26 | 0.73 (0.45; 1.20) | 7 | 0.49 (0.18; 1.31) |  | 47 | 0.94 (0.61; 1.44) | 6 | 1.00 (0.41; 2.44) |

[TSH: thyroid stimulating hormone; fT3: free triiodothyronine; fT4: free thyroxine]

^*^p≤0.05

Supplementary Table. Adjusted odds ratio of confirmed and suspected ADHD cases outside the age-specific reference intervals (2.5th – 97.5th percentile) defined by Kratzsch et al. for TSH, fT3, and fT4. Data expressed as adjusted odds ratio (OR) with 95% Confidence Interval (CI). Models have been adjusted for sex, age, and weight at birth, and the following covariates: mother’s smoking habit during gestation and the current BMI z-score of the child/adolescent.
